# Assessment of Internal Damage in Sandwich Structures by Post-Processing of Mode Shapes Using Curvelet Transform

**DOI:** 10.3390/ma14164517

**Published:** 2021-08-11

**Authors:** Andrzej Katunin, Sandris Ručevskis

**Affiliations:** 1 Department of Fundamentals of Machinery Design, Faculty of Mechanical Engineering, Silesian University of Technology, Konarskiego 18A, 44-100 Gliwice, Poland; 2 Institute of Materials and Structures, Riga Technical University, Kipsalas Iela 6A, LV-1048 Riga, Latvia; sandris.rucevskis@rtu.lv

**Keywords:** damage identification, damage quantification, sandwich structures, modal analysis, curvelet transform, standardized damage index

## Abstract

Identification and quantification of structural damage is one of the crucial aspects of proper maintenance of mechanical and civil structures, which is directly related to their integrity and safety. The paper presents a novel approach for detecting various types of damage in sandwich structures by processing the mode shapes using a hybrid algorithm based on the curvelet transform and the standardized damage index concept. The proposed approach uses the properties of directional selectivity, absence of the boundary effect, typical of such a class of transforms, and excellent filtration capabilities of the curvelet transform as well as the classification hypothesis in the standardized damage index, which allows the exclusion of irrelevant information and emphasizes proper damage location and shape. The proposed hybrid algorithm allowed to successfully identify a subsurface core damage in sandwich structures, such as local lack of a core or its debonding from facings. The performed quantification study aimed to evaluate the correctness of identified damage shape confirmed the validity and accuracy of the proposed algorithm not only for the damage detection and localization but also for the estimation of the size of structural damage.

## 1. Introduction

Sandwich composite structures have found wide application in numerous civil and mechanical structures, offering great performance in terms of their strength and stiffness and simultaneously being lightweight, which makes it possible to significantly reduce a mass of construction without losing its mechanical properties [1,2,3,4]. In spite of the excellent mechanical performance in terms of a possibility of carrying significant loading of various types, sandwich structures are susceptible to defects occurring during the manufacturing process, such as debonding, defects of a core, buckling defects, etc., as well as operational damage, such as low-velocity impact damage [1,5]. Such defects and damage may significantly decrease the mechanical properties of a sandwich structure, causing a drop of stiffness, which, in turn, could be an initiator of the development of a failure mechanism, causing final failure and disintegration. Therefore, defect and damage identification is one of the crucial tasks during the operational inspection of sandwich structures.

Recently, numerous non-destructive testing (NDT) techniques have been adapted to detect, localize, and identify surface and internal flaws in sandwich structures. These techniques, depending on the industrial conditions, in which a sandwich structure is operated, include visual testing [6], ultrasonic testing [7,8,9], thermography [10,11], eddy-current testing [12], shearography [7], guided waves-based techniques [1,13,14], electrical impedance [15], terahertz testing [16], etc. as well as by various embedded sensors in smart structures [17]. An overview of defects and damage detectability in sandwich structures can be found e.g., in the review paper of Nsengiyumva et al. [18]. However, the above-mentioned techniques are usually demanding in setup preparation and are complex in operation as well as costly. In spite of this, the vibration-based damage identification techniques with a possibility of high-precision measurements (e.g., using laser Doppler vibrometers (LDVs) or Digital Image Correlation (DIC) measurement technique) and appropriate processing of testing results can provide a good and cost-effective alternative for defect and damage identification in sandwich structures.

Vibration-based damage identification methods are usually based on the processing of mode shapes or modal curvatures acquired from the vibrating structures. This is because, from the parameters, which can be acquired from a modal analysis (i.e., natural frequencies, and corresponding mode shapes and damping factors), the mode shapes and their derivative representations are the most sensitive to structural damage. Moreover, analysis of mode shapes gives a possibility to not only detect damage but determine its spatial location and sometimes also a type and other properties of damage. Another great advantage of this approach is the possibility of damage identification without a baseline, i.e., data from a healthy structure or its model is not necessary for detecting damage, the damage identification procedure is performed solely on a tested (damaged) structure. This is possible due to the presence of local changes in structural properties, which also affect the modal response locally. Consequently, to enhance the detectability of damage, most processing algorithms are applied to mode shapes.

Among the considered processing algorithms, one can distinguish two main groups: the algorithms based on various types of damage indices (DIs) and those based on space-frequency analysis. The first group covers numerous approaches based on energy operators [19,20,21,22], curvature mode shape differences [23,24], modal criteria and parameters [5,25,26,27], and many other. The second group implies a direct application of filtering procedure to mode shapes or modal curvatures using the algorithms primarily based on wavelet transforms (WTs) [28,29] and hybrid algorithms using them [30,31]. As it was shown in numerous previous studies (see e.g., [32,33]), the effectiveness of WTs in damage identification problems is significantly influenced by the properties of a selected wavelet, i.e., its type, number of vanishing moments, compactness, power distribution, etc. This, in turn, has a direct influence on the filtering ability, since WTs, in particular their discrete representations, can be considered as a set of low- and high-pass filters. Unfortunately, the selection of a proper wavelet basis function is not guided by any standard rules, and in many cases, this selection is performed by the trial-and-error method. Additionally, the two-dimensional (2D) wavelets are unable to represent properly the curvilinear damage signatures, since at each level of decomposition they operate on a square grid [34]. This means that in the case of the existence of damage with a curved boundary, it would not be possible to track its shape precisely. Another problem appearing during the application of WTs is the presence of the boundary effect, causing a significant increase of the resulting wavelet coefficients in the vicinity of borders of a tested structure. To overcome these deficiencies, some non-parametric methods were applied for damage identification, including cross-correlation [35], Vigner-Wille distribution [36], and S-transform [37]. However, a problem with properly filtering out the damage signatures from processed mode shapes or curvatures remains open in the area of structural damage identification. Fortunately, the above-mentioned problems with the boundary effect, ineffective filtering, and some other directly related to the classical tensor-product construction of 2D wavelet bases, like poor directional selectivity or non-smooth windowing [38], are resolved by the family of X-let transforms being developed over the last 20 years and currently containing hundreds of application-oriented transforms with enhanced properties with respect to classical WTs.

Like in the case of many other filtering approaches, the origin of X-lets is the image processing, where the filtration ability together with a proper representation of features of a processed image, such as discontinuities, artifacts, smooth edges, etc., are the key properties of the applied tools in numerous applications, primarily in processing of biological and medical images. Further, due to their proven advantageous properties, the X-lets started to be applied in numerous applications in technical sciences, including mechanical and civil engineering applications, and, in particular, quality assurance, NDT, and structural damage identification. Mumtaz et al. [39] used contourlets for tracking cracks from images of aircraft structures. Xu et al. [40] used shearlets for the classification of surface defects of machined elements, similarly to Umamaheswara Raju et al. [41], who used curvelets for an evaluation of a surface quality after machining. The filtering approach based on the curvelet transform (CT) was proposed by Tzanis in [42], where he applied it for fracture detection in ground probing radar data. Anandan and Sabeenian [43] used CT for the detection of fabric defects. There were also a few applications of X-lets in damage identification problems. Bagheri et al. [44,45] used CT for identification of simulated structural damage in plates using their mode shapes, De Marchi et al. [46] used curvelets in their structural health monitoring approach for improvement of a damage detectability in a guided wave-based technique, while the recent studies of Vafaie and Salajegheh [47] proposed an application of wavelets and contourlets for vibration-based damage identification in plates.

The lack of systematic studies on the application of X-let transforms (XTs) in damage identification means that this problem remains open. Moreover, the variety of transforms within the XTs family raises the question of a suitable transform for damage identification. The previous comparative studies on the performance of wavelets vs. X-lets [47,48] clearly show significant improvement of filtering ability and sensitivity to damage of the latter ones, while the comparisons of the performance of X-lets between each other [44,47,48] indicates that curvelets provide the best filtering performance, which makes them suitable for improvement of sensitivity to damage in damage identification problems.

The aim of this study is to analyze damage detectability in sandwich composite plates with simulated damage based on the processing of their mode shapes using CT and standardized damage index (SDI) concept and to highlight its advantages not only in filtering performance, but also in the evaluation of a shape of damage due to enhanced directional selectivity of CT. Thanks to merging the CT-based algorithm with SDI determination in the second step of processing, precise quantification of shapes of the considered damage in sandwich plates is possible.

## 2. Tested Structures and Damage Identification Algorithm

Sandwich structures are specific composites that have found application in numerous mechanical and civil constructions. Their primary advantage, namely a very high stiffness-to-mass ratio, defines the type of the most critical damage for these structures, like damage of a core or debonding between a core and facings. These damage scenarios were simulated in the tested plates and are described in detail in Section 2.1. In Section 2.2 the testing setup and results of the modal analysis are presented. Then, the fundamentals of the CT and its advantages over other transforms of a similar type are presented in Section 2.3, and its incorporation in the damage identification algorithm with additional post-processing is the subject of Section 2.4.

### 2.1. Pre-Damaged Sandwich Structures

The tested sandwich structures with the spatial dimensions of 300 × 300 mm and a total thickness of 4.1 mm are composed with a honeycomb core made of Nomex^®^ (DuPont, Wilmington, DE, USA) aramid paper saturated with a phenolic resin with a thickness of 3 mm and two facings made of glass-fiber reinforced polymer (GFRP) composite with a thickness of 0.6 mm each (the decrease of a total thickness is a result of a manufacturing process). The core has a density of 29 kg/m^3^ and a diameter of a single cell of 2.5 mm. The GFRP facings were designed as intentionally transparent, which made it possible to see the shape and location of simulated internal damage with a naked eye. More details on constituents used for manufacturing the plates can be found in [28]. According to the performed quasi-static tests [49], the tensile strength for this structure equals 21.45 MPa, while the buckling strength limit in the three-point bending testing mode equals 52.61 MPa.

**Figure 1:**
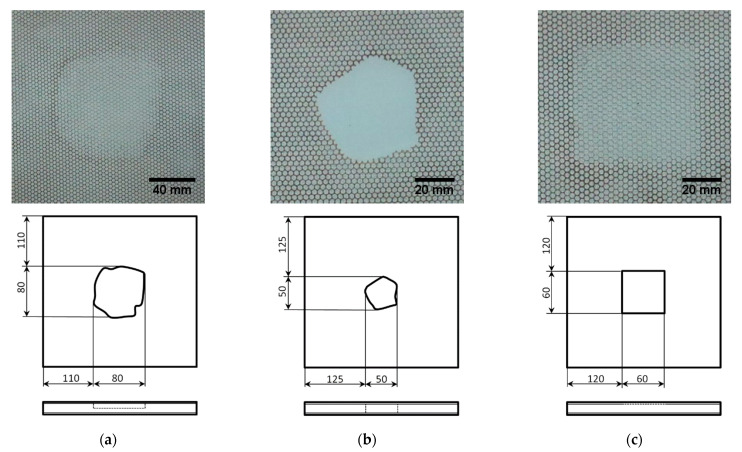
The images and schemes of the damaged sandwich plates: (**a**) partial local lack of the core {1}, (**b**) full local lack of the core {2}, (**c**) debonding between the core and the upper facing {3} [28,37] with permission from Elsevier.

The sandwich plates were manufactured and supplied by the PPHU Surfpol (Rawa Mazowiecka, Poland) in the vacuum-assisted resin transfer molding manufacturing technique and simulated damage of a core was introduced at the manufacturing stage. Three damaged plates were considered in this study. They consisted of the partial local lack of the core with an irregular boundary placed in the center of the plate (the thickness of a core in this location was reduced twice) {1}, the full local lack of the core with irregular boundary placed in the center of the plate {2}, and the debonding between the core and the upper facing with a square shape and dimensions of 60 × 60 mm placed in the center of the plate {3}. The images of the damaged plates together with their schemes and dimensions are presented in Figure 1.

### 2.2. Acquisition of Mode Shapes

**Figure 2:**
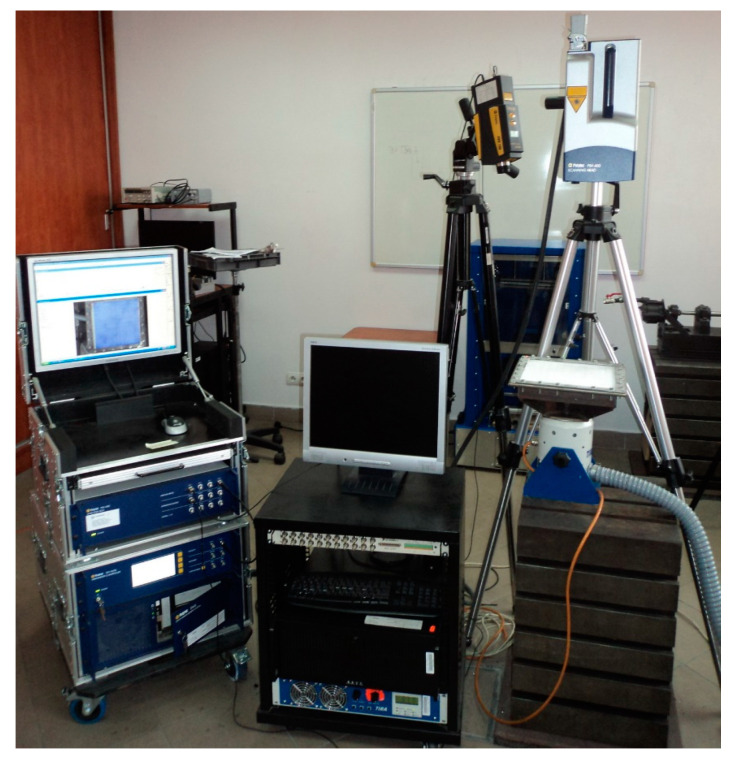
The experimental setup used for the acquisition of mode shapes [28] with permission from Elsevier.

The modal analysis was performed for the fully clamped specimens described in Section 2.1 using two LDVs. The tested structures were clamped in a steel square frame with 24 bolts on its perimeter using the Jonnesway^®^ (Taipei, Taiwan) T07030N dynamometric wrench to ensure the same clamping conditions and simultaneously do not affect the structural properties due to clamping. The clamping resulted in a reduction of a scanning area to 250 × 250 mm. In scenarios {1} and {3}, the damage sites were located close to the scanned side of the plates. Prior to testing, the upper surfaces of the tested structures were covered with the Helling^®^ (Heidgraben, Germany) anti-glare spray to ensure the proper reflection ability of a laser beam of LDV. Then, in the Polytec^®^ (Waldbronn, Germany) dedicated software, 64 × 64 equidistant measurement points were defined in a scanning area. The frame was mounted on the TIRA^®^ (Schalkau, Germany) TV-51120 electrodynamic shaker, connected with the TIRA^®^ BAA 500 power amplifier, which was used for vibration excitation. The scanning LDV Polytec^®^ (Waldbronn, Germany) PSV-400, connected to the vibrometer controller Polytec^®^ (Waldbronn, Germany) OFV-5000 with the built-in velocity decoder and to a PC, was used for a measurement of a vibration velocity in the defined grid of measurement points, while the point LDV Polytec^®^ (Waldbronn, Germany) PDV-100 was focused on a clamping frame to acquire the reference signal, which made it possible to separate vibrations of a frame from vibrations of a whole system, which, in turn, allowed acquiring vibration of the tested structures. The experimental setup is presented in Figure 2.

**Figure 3:**
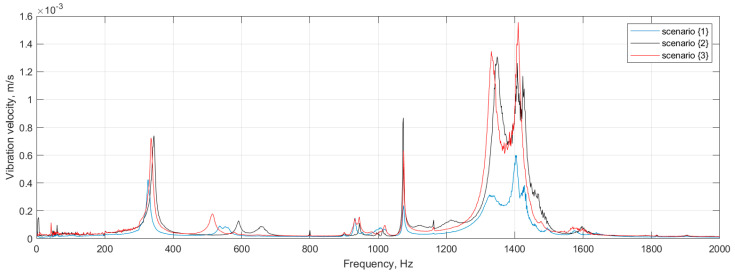
FRFs of the tested sandwich structures.

**Figure 4:**
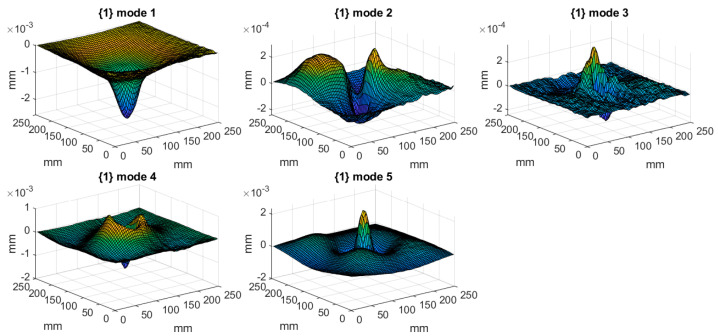
The considered mode shapes for the plate with a partial local lack of the core.

**Figure 5:**
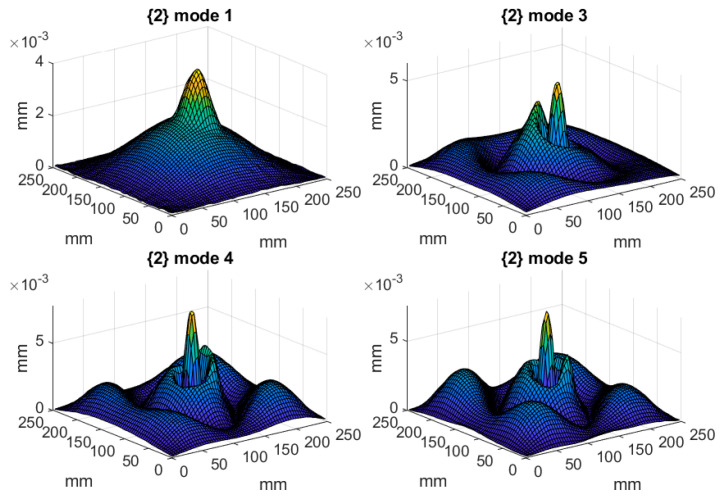
The considered mode shapes for the plate with a full local lack of the core.

**Figure 6:**
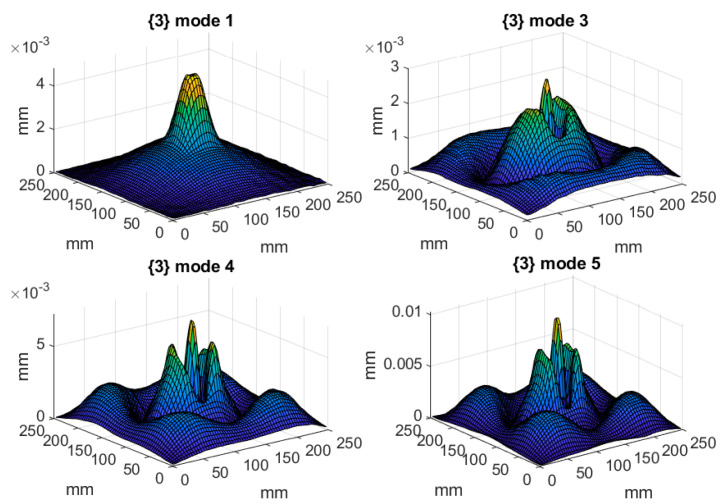
The considered mode shapes for the plate with a debonding between the core and the upper facing.

The tested structures were excited with a pseudo-random signal in the frequency band of 0–2000 Hz with a frequency resolution of 1.25 Hz to excite all eigenfequencies in the defined frequency band. To increase the accuracy of the obtained results, five measurements were performed in each defined point and the acquired signals were averaged. Since the magnitude of vibration is directly related to the damage detectability, the following rule was applied: a given mode shape was taken into consideration if its magnitude in the frequency response function (FRF) was at least 20% of the magnitude of the highest observed peak in FRF. This assumption was made due to the presence of measurement noise in signals, and the assumed threshold was determined empirically based on preliminary analysis. After applying this threshold, the mode shapes significantly biased by measurement noise were excluded from consideration, since due to the high level of noise the detectability of damage using these mode shapes was very low. The resulting FRFs of the tested structures are presented in Figure 3, while the eigenfrequencies and the corresponding mode shapes for the tested structures are presented in Table 1 and Figure 4, Figure 5 and Figure 6, respectively. As it can be observed in Table 1, due to small differences between the determined eigenfrequencies, even detection of structural damage based on analysis of changes in eigenfrequencies can be difficult, which additionally justifies the necessity of effective post-processing of mode shapes.

### 2.3. Acquisition of Mode Shapes

CT developed by Candès et al. [50] is the multiscale transform with numerous improved properties with respect to traditional wavelet-based multiscale representations. The main advantages of CT cover multidirectionality, thanks to the construction of the pyramidal representation across the scales, a possibility of effective detection of curved anomalies in a signal, and the enhanced filtering capabilities. All of them are of crucial importance in damage identification problems and have a significant influence on damage detectability, which is demonstrated in the next section.

The family of curvelets considered in this transform as basis function are defined as follows [51]:

(1) $$\varphi_{j,l,k}\left(x\right)=\varphi_{j}\left(R_{\theta_{l}}\left(x-x_{k}^{\left(j,l\right)}\right)\right),$$

where $x_{k}^{\left(j,l\right)}=R_{\theta_{1}}^{-1}\left(2^{-j}k_{1},2^{-j/2}k_{2}\right)$ defines the radial position, and $R_{\theta }=\left(\begin{array}{cc} \cos\theta & \sin\theta \\ -\sin\theta & \cos\theta \end{array}\right)$ defines the angular position of a curvelet at scales of $2^{j}$. The curvelet coefficients c(·) are defined by the inner product of a curvelet and a function f(x):

(2) $$c\left(j,l,k\right)=\int_{{\mathbb{R}}^{2}}^{}f\left(x\right)\varphi_{j,l,k}\left(x\right)dx,\;f\in L^{2}\left({\mathbb{R}}^{2}\right).$$

As one can see, CT is defined in polar coordinates, and the decomposition process is performed using a pair of the window functions: radial W(r) and angular V(r), subject to the admissibility conditions:

(3) $$\overset{\infty }{\underset{j=-\infty }{\sum }}W^{2}\left(2^{j}r\right)=1,\;r\in \left(\frac{3}{4},\frac{3}{2}\right),$$

(4) $$\overset{\infty }{\underset{l=-\infty }{\sum }}V^{2}\left(t-l\right)=1,\;t\in \left(-\frac{1}{2},\frac{1}{2}\right),$$

which lead to the frequency window defined in the Fourier domain as follows [50]:

(5) $$U_{j}\left(r,\theta \right)=2^{-3j/4}W\left(2^{-j}r\right)V\left(\frac{2^{\left\lfloor j/2\right\rfloor }\theta }{2\pi }\right),$$

where ⌊·⌋ denotes integer. Since CT operates in the frequency domain, it is essential to present (2) in terms of frequency ω:

(6) $$c\left(j,l,k\right)=\frac{1}{4\pi^{2}}\int^{\;}\hat{f}\left(\omega \right)U_{j}\left(R_{\theta_{l}}\omega \right)\exp\left(i\langle x_{k}^{\left(j,l\right)},\omega \rangle \right)d\omega ,$$

where $\hat{\cdot }$ denotes the frequency domain. The representation can be easily transformed into Cartesian coordinates, making it useful for data processing in the form of 2D matrices. Following this, Equation (6) takes a form [50]:

(7) $$c\left(j,l,k\right)=\frac{1}{4\pi^{2}}\int^{\;}\hat{f}\left(S_{\theta_{l}}\omega \right){\tilde{U}}_{j}\left(\omega \right)\exp\left(i\langle 2^{-j}k_{1},2^{-j/2}k_{2},\omega \rangle \right)d\omega ,$$

where $S_{\theta }=\left(\begin{array}{cc} 1 & 0\\ -\tan\theta & 1 \end{array}\right)$ is the shear matrix, and ${\tilde{U}}_{j}\left(\omega \right)$ is the frequency window in the Cartesian coordinate system, which changes its form from (5) to the following:

(8) $${\tilde{U}}_{j}\left(\omega \right)={\tilde{W}}_{j}\left(\omega \right){\tilde{V}}_{j}\left(\omega \right),$$

where

(9) $${\tilde{V}}_{j}\left(\omega \right)=V\left(2^{\left\lfloor j/2\right\rfloor }\frac{\omega_{2}}{\omega_{1}}\right),$$

(10) $${\tilde{W}}_{j}\left(\omega \right)=\sqrt{\Phi_{j+1}^{2}\left(\omega \right)-\Phi_{j}^{2}\left(\omega \right)},\;j\geq 0.$$

In (9) and (10), $\Phi_{j}\left(\omega_{1},\omega_{2}\right)=\varphi \left(2^{-j}\omega_{1}\right)\varphi \left(2^{-j}\omega_{2}\right)$ is the product of the 1D windows, 0≤φ≤1, $\varphi =\{\begin{array}{cc} 1, & \left[-\frac{1}{2},\frac{1}{2}\right]\\ 0, & \left[-2,2\right] \end{array}$, $2^{j}\leq \omega_{1}\leq 2^{j+1}$, $-2^{-j/2}\leq \frac{\omega_{2}}{\omega_{1}}\leq 2^{-j/2}$.

The discrete version of CT, called by the authors of [50] the digital CT, changes the expression (7) to the following form:

(11) $$c\left(j,l,k\right)=\underset{n_{1},n_{2}}{\sum }\hat{f}\left[n_{1},n_{2}-n_{1}\tan\theta_{l}\right]{\tilde{U}}_{j}\left[n_{1},n_{2}\right]\exp\left(i2\pi \left(\frac{k_{1}n_{1}}{L_{1,j}}+\frac{k_{2}n_{2}}{L_{2,j}}\right)\right),$$

which is one of two possible methods of computing the coefficients called the digital CT via wrapping. More details on the theoretical basis of CT can be found in [50,51,52].

### 2.4. Damage Identification Algorithm

The damage identification algorithm was based on the discrete CT implemented by the Curvelet.org team in the form of the CurveLab Toolbox for Matlab^®^ (version 2020b, MathWorks, Natick, MA, USA) The curvelet coefficients were calculated according to (11) for all considered mode shapes (see Figure 4, Figure 5 and Figure 6) of the tested structures, and the obtained values were raised to the power of 2 in order to separate insignificant low-value coefficients. In the following study, CT via wrapping was used. According to [51], obtaining the coefficients c(j,l,k) consists of four steps:

(1) Calculating 2D Fourier transform (FT) to transform space domain variables f[s,p] (mode shapes, in our case) in the form of the frequency domain variables $\hat{f}\left[n_{1},n_{2}\right]$.
(2) Calculating the product of the determined variables with a frequency window: $\hat{f}\left[n_{1},n_{2}\right]{\tilde{U}}_{j}\left[n_{1},n_{2}\right]$.
(3) Wrapping the above product around the origin: ${\tilde{f}}_{j,l}\left[n_{1},n_{2}\right]=W\left(U_{j,l}\hat{f}\right)\left[n_{1},n_{2}\right]$.
(4) Calculating inverse 2D FT for ${\tilde{f}}_{j,l}$ to obtain c(j,l,k).

In practical situations, experimental mode shapes are inevitably biased by measurement noise, which can cause local perturbations in curvelet coefficients. These perturbations could be mistakenly interpreted as damage or they could mask the peaks induced by damage, and thus, lead to false-positive or false-negative damage identification. To overcome this problem, it was proposed to define DI as the summation of the curvelet coefficients for all modes normalized with respect to the largest magnitude value of each mode as follows:

(12) $$\mathrm{DI}\left(s,p\right)=\overset{M}{\underset{m=1}{\sum }}\frac{c{\left(s,p,m\right)}^{2}}{c{\left(m\right)}_{\max}^{2}},$$

where s and p are the coordinates of DIs corresponding to the grid of measurement points, and m stands for the mode number.

The principal goal of the damage identification algorithm was to provide useful information about damage in the sandwich structures by quantitatively evaluating the obtained results after the application of CT. In recent years, statistical interference methods, such as estimation and hypothesis testing have been widely used in the field of damage identification for the evaluation of the mode shape data, among other purposes [33,53]. In this study, a statistical hypothesis testing scheme was followed to classify damaged and healthy elements based on the obtained CT results and pre-defined damage threshold values.

In the first step of the hypothesis testing, the DIs for each element calculated according to (12) were standardized in order to obtain SDI:

(13) $$\mathrm{SDI}\left(s,p\right)=\frac{\mathrm{DI}\left(s,p\right)-\mu_{\mathrm{DI}}}{\sigma_{\mathrm{DI}}},$$

where $\mu_{\mathrm{DI}}$ and $\sigma_{\mathrm{DI}}$ are the mean and the standard deviation of DIs, respectively.

The next step was to define the null and alternative hypotheses for the classification of the elements:

- *H*_0_—the element (*s,p*) of a structure is healthy;
- *H*_1_—the element (*s,p*) of a structure is damaged.

In general, rejection of the null hypothesis *H*_0_ in the test indicates the presence of damage in the element and vice versa.

The probability density of SDIs that is obtained from a mode shape of a healthy structure would usually have a normal distribution. On the other hand, the probability density of SDI is expected to have non-normal distribution with extreme values in the tails of the density distribution due to a damage presence in a structure. Therefore, by assuming that SDI is normally distributed, the probability density function may be used to classify elements by employing a one-tailed hypothesis test [53]:

(14a) $$H_{o}:\mathrm{SDI}\left(s,p\right)<C_{r},$$

(14b) $$H_{1}:\mathrm{SDI}\left(s,p\right)>C_{r}.$$

**Figure 7:**
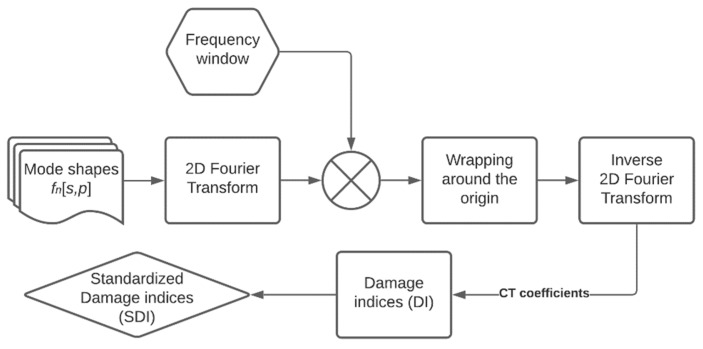
The steps of processing the mode shapes according to the developed damage identification algorithm.

The objective of the hypothesis testing is to decide whether the elements of a structure are damaged based on the significance level—a pre-designed probability threshold, above which the null hypothesis will be rejected. The most commonly used threshold values $C_{r}$ for the damage identification include 1.28, 2, and 3, which corresponds to 90%, 95%, and 99% confidence levels for the presence of damage, respectively [33,53]. In the final step, the threshold value for the 90% confidence level was selected to classify damaged elements, which resulted from the initial analysis of considered data. For clarity, the above-described damage identification algorithm is presented in the form of a flowchart in Figure 7.

This assumed threshold value provides an advantageous tradeoff between the accuracy of damage detection and localization and the level of noise. The values of SDIs lower than the threshold value of 1.28 were set to zero to filter out smaller peaks, which are not associated with damage.

## 3. Results and Discussion

The acquired mode shapes of the tested sandwich plates were processed according to the algorithm presented in Section 2.4. The obtained results were focused on the detectability of simulated damage as well as their quantification in terms of their planar shapes, which is presented below.

### 3.1. Analysis of Detectability of Various Damage Types

**Figure 8:**
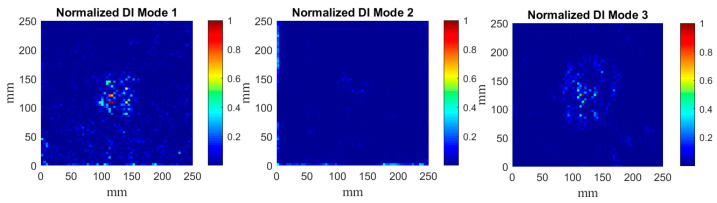

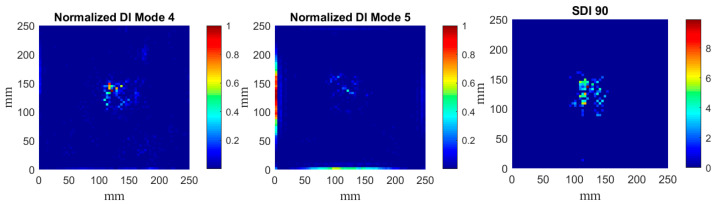
The determined normalized DIs for the mode shapes for the plate {1} and SDIs with the 90% confidence level.

**Figure 9:**
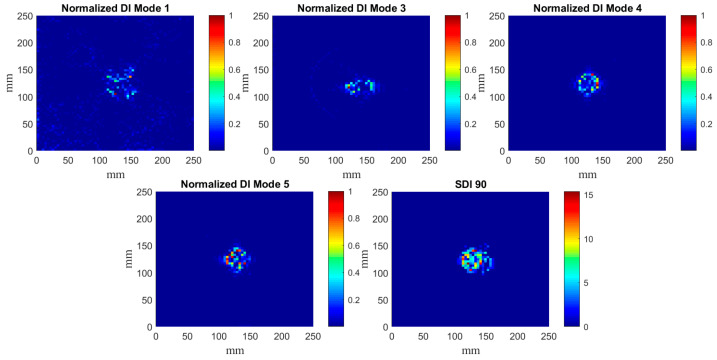
The determined normalized DIs for the mode shapes for the plate {2} and SDIs with the 90% confidence level.

**Figure 10:**
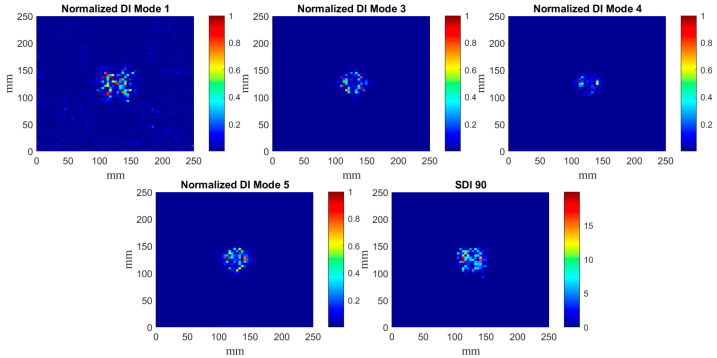
The determined normalized DIs for the mode shapes for the plate {3} and SDIs with the 90% confidence level.

The determined DIs according to the algorithm presented in Section 2.4 for particular mode shapes are presented in Figure 8, Figure 9 and Figure 10. According to the results of the previous studies [33], the confidence level for SDI was assumed as 90%, which resolves a compromise between measurement and processing noise and the true-positive DIs representing the damage signatures.

The obtained DIs clearly show that the considered damage scenarios are detectable in every considered case, however, the planar shapes of damage signatures differ from each other for various mode shapes, which can be explained by the variability of local magnitudes of mode shapes and their direct influence on the curvelet coefficients.

It can be noticed that CT demonstrates excellent performance in the filtering of the raw mode shapes obtained from measurements, i.e., in most cases only the damage signatures are visible. Comparing the results presented in Figure 8, Figure 9 and Figure 10 with the previous results obtained with the fractional discrete WT [28] and with optimization of its WT’s parameters [30], one can conclude about good detectability of damage together with an enhanced resolution of CT with respect to the previously applied WT. Comparing the obtained results to those reported in [28,30] one can observe that the proposed algorithm allows for more accurate mapping of a damage shape than the WT-based algorithms used previously. However, in contrast to these WT-based algorithms, the algorithm proposed in this study slightly underestimates the true damage extent. This underestimation is a result of an application of the second step of processing, namely, the SDI procedure. However, the applied SDI procedure (see Section 2.4 for details) allowed to reduce the remaining measurement and processing noise in the resulting sets of DIs, which made it possible to prepare the obtained results for the next processing step, namely the quantification of the shapes of damage signatures. The application of the SDI procedure is a compromise between the proper mapping of a true damage shape and filtering out measurement noise that can be considered as a false indicator of damage. It is also worth mentioning that CT did not produce the boundary effect, i.e., the extremely high values of coefficients at the boundaries of a signal due to the translation of a basis function on this signal, typical for most of WTs.

The calculated DIs for the plate with a partial local lack of the core (Figure 8) confirms the suitability of the proposed SDI procedure for the pursuit of the development of an independent and autonomous damage identification algorithm. One can see that the resulting SDI plot clearly indicates the damage location, while individually only DIs for 3 out of 5 considered mode shapes were able to detect damage and correctly point out its location when a 90% confidence level was selected to classify elements. These results emphasize the necessity to normalize DIs for particular mode shapes in order to obtain equally weighted resulting sets of DIs. This way, the risk of few corrupted measurement data sets causing false or missed damage identification is significantly reduced. Another advantage of the present SDI procedure highlighted by Figure 8, is that mode shape transformation data can simply be fed to the algorithm, and the decision on whether any element of the structure is damaged is acquired automatically based on the selected damage threshold value. Hence, the damage identification algorithm can operate autonomously by minimizing the engagement of the data interpreter in the process.

### 3.2. Quantification of Damage Shape

**Figure 11:**
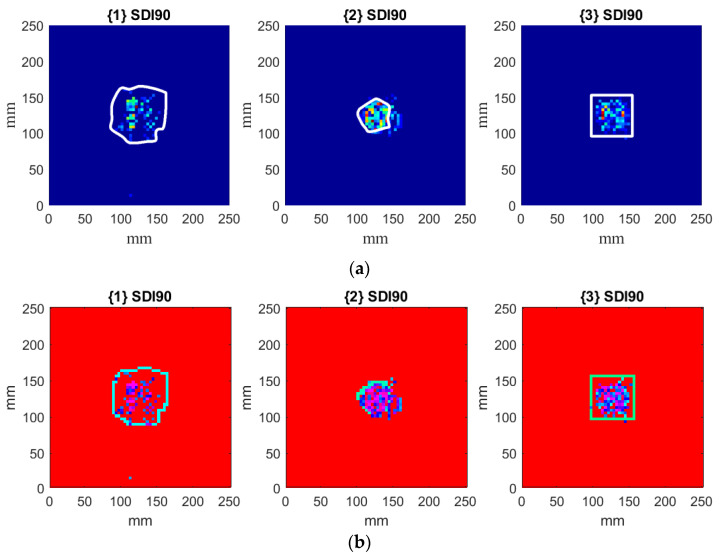
The results of merging of the boundaries of damage with the identified damage for the considered scenarios (**a**) and with the discretized version of the true boundaries of damage (**b**).

To compare the obtained results with the true shapes of damage in the considered damage scenarios, the photographs of damaged sandwich plates presented in Figure 1 were subjected to initial image processing, which was performed to retrieve the true shapes of damage. Firstly, the contours of damage were obtained using boundary detection techniques, and the detected boundaries were appropriately scaled to match true dimensions. Then, the boundaries were transformed to the sets of coordinates of points representing these boundaries and merged with the identified damage for each considered scenario (see Figure 11a). Finally, the resulting sets of coordinates of the detected boundaries were discretized in order to match the defined grid of 64 × 64 measurement points. The results of merging the discretized boundaries with the identified damage are presented in Figure 11b. The colors on the presented plots were selected in such a way as to obtain good contrast between the identified SDIs and the boundaries of true damage.

As it can be observed, the identified damage corresponds to the true boundaries of damaged regions, however, some misestimations are observable. In particular, the underestimation of damage areas is noticed for scenarios {1} and {3}, while an overestimation is observable for scenario {2}. The observed misestimations may appear due to several reasons: specific location of nodal lines of the considered mode shapes, which made it impossible to obtain positive SDIs in some locations due to low vibration magnitude in these locations; processing procedures, like filtering of CT and rejection of SDIs following the hypotheses (14). Nevertheless, the qualitative analysis of the obtained results shows a good correspondence of the identified damage with the boundaries of true damage for all considered scenarios.

**Figure 12:**
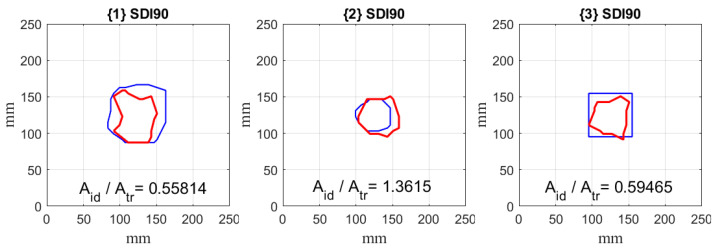
The quantification results for the considered damage scenarios with the ratios of areas of identified *A_id_* and true damage *A_tr_*.

To assess the accuracy of damage quantification quantitatively, the envelopes for identified positive SDIs were calculated. The envelopes were controlled by the shrink factor, which is a scalar value in the range of [0, 1], and takes the value of 1 for a convex hull and the value of 0 for the tight boundary. In these limit cases, one can observe overestimation in the case of application of a convex hull, and underestimation of the results in the case of a tight boundary. After preliminary testing, the value of a shrink factor was assumed as 0.5, which provides a tradeoff between the mentioned limit cases. The obtained results with the determined ratios of the damage areas for the considered damage scenarios are presented in Figure 12.

From the quantification results, one can observe that the identified damage matches well the true boundaries of damage, which confirms the effectiveness of the proposed algorithm in this study. The quantitative analysis resulted in differences in the true and identified damage areas not exceeding 50%, which is comparable with results of quantification, obtained by other NDT methods, e.g., ultrasonic testing in the C-Scan mode.

The obtained results make it possible to formulate a statement that the proposed algorithm can be successfully used in a larger class of damage identification and quantification problems, including various types of damage and structures containing them.

## 4. Conclusions

The effective hybrid algorithm based on CT and SDI concept, which combines the advantages of CT, namely directional selectivity and excellent filtration ability, and the advantages of SDI, such as selection of only relevant information on damage, is proposed in this study. The algorithm was tested on mode shapes of sandwich structures with various types of damage obtained experimentally during modal analysis testing. The results show a great sensitivity of CT to these types of damage, which resulted in the identification of all considered damage scenarios with higher accuracy in comparison to the previously applied classic WT-based algorithms. The application of the SDI concept improved the quantification of damage sufficiently by discarding the irrelevant curvelet coefficients that do not represent damage. The results of quantification of subsurface damage in the tested sandwich structures show high validity, i.e., in all scenarios the error in comparison with the boundaries of the true damage not exceeding 50%, which is comparable with numerous NDT methods, like ultrasonic testing, widely applied in structural inspections.

Based on the results of the performed studies, it can be concluded that using the proposed approach one can effectively identify and quantify subsurface damage in sandwich structures. The proposed algorithm can be applied to numerous other structural damage identification problems, especially when damage is small, or measurement data is biased by measurement and/or processing noise.

## Figures and Tables

**Table 1:** The eigenfrequencies of the tested sandwich structures.

Scenario	Type of Damage	Mode 1, Hz	Mode 2, Hz	Mode 3, Hz	Mode 4, Hz	Mode 5, Hz
{1}	Partial local lack of the core	326	932	1075	1326	1405
{2}	Full local lack of the core	350		1073.75	1355	1427.5
{3}	Debonding between the core and the upper facing	335		1075	1331.25	1410

## Data Availability

The data that support the findings of this study are freely available as a part of the WavStructDamAs benchmark at the website http://kpkm.polsl.pl/wavstructdamas (accessed on 22 June 2021).
